# Chronic Toxicity Study of Neosaxitoxin in Rats

**DOI:** 10.3390/md12095055

**Published:** 2014-09-25

**Authors:** Ramiro J. Zepeda, Manila Candiracci, Nicolas Lobos, Sebastian Lux, Hugo F. Miranda

**Affiliations:** 1 Molecular and Clinical Pharmacology Program, Institute of Biomedical Sciences, Medical School, University of Chile, Independencia 1027, 8380453 Santiago, Chile; E-Mails: ni_lobos@med.uchile.cl (N.L.); slux@med.uchile.cl (S.L.); hmiranda@med.uchile.cl (H.F.M.); 2 School of Medicine, ICBM, Medical School, Universidad de Chile, Independencia 1027, 8380453 Santiago, Chile; 3 Anesthesia Department, Brigham and Woman’s Hospital, Harvard University, 75 Francis Street, Boston, MA 02115, USA; E-Mail: mcandiracci@partners.org

**Keywords:** neosaxitoxin, marine toxin, chronic toxicity, acute toxicity

## Abstract

Neosaxitoxin (NeoSTX) is a specific reversible blocker of voltage gated sodium channels on excitable cells. In the last decade, it has been tested in a number of interesting clinical trials, however there is still little information available on mammalian toxicity. Rats were treated for 12 weeks with doses of 1, 3 or 6 μg/kg of subcutaneous NeoSTX. At weeks 12 and 17, animals were sacrificed and blood samples collected for hematological and biochemical analysis. Organs were harvested for weight determination and histopathological assessments. The lowest acute toxicity via the intraperitoneal (ip) route was (30.35 μg/kg) and there was no significant difference between intramuscular and subcutaneous routes (11.4 and 12.41 μg/kg). The NeoSTX adiministration did not produce lethality at week 12 and after five weeks of suspension. NeoSTX 6 μg/kg ip produced reductions (*p* < 0.05) in body weight and food intake, and increased blood level of total and direct bilirubin, GGT and SGOT at week 12; all of these were reversed in the recovery period. NeoSTX 1 and 3 μg/kg ip did not show significant changes with the control group. Histopathological presentations were normal in all groups. This study revealed that NeoSTX is safe *in vivo*, giving a reliable security margin for its use like a local anesthetic.

## 1. Introduction

Paralytic shellfish toxins (PST) is a group of alkaloid neurotoxins with high affinity for voltage-dependent sodium channels, which causes muscle paralysis by blocking the nervous impulse [1,2]. Up to now, over 57 isomers related to this group of PST have been identified, each of them possessing a different affinity with sodium channels and toxic capabilities [3,4]. PST are produced by marine dinoflagellates of the *Alexandrium*, *Pyrodinium* and *Gymnodinium* genres, and also by certain species of cyanobacteria [5,6].

NeoSTX-1*H*,10*H*-Pyrrolo(1,2-*c*)purine-10,10-diol,2-amino-4((aminocarbonyl)oxy)methyl-3a,4,5,6,8,9-hexahydro-5-hydroxy-6-imino-(3a*S*(3aalpha,4alpha,10aR*)), is a PST-toxin with the molecular formula C_10_H_18_N_7_O_5_ (Molecular Weight = 315 g/mol) and is composed of a 3,4-propinoperhydropurine tricyclic system. NeoSTX belongs to the large family of guanidinium-containing marine natural products, due to the presence of two guanidine groups which are responsible for its high polarity [2]. Its effect is recognized as powerful and potentially lethal due to its affinity to voltage-gated sodium channels’ blocking, thus, preventing the transmission of nerve impulses transmitted through susceptible fibers [3,7]. This is mediated by the interaction between the positively charged guanidinium groups of NeoSTX with negatively charged carboxyl groups at site 1 of the Na^+^ channel, thereby blocking the pore. Moreover, its feature and similarity to Tetrodotoxin (TTX) support its pharmacodynamic, it has been assessed in several studies as a local anesthetic [8].

Classic local anesthetics, such as bupivacaine, lidocaine and proparacaine, can be infiltrated subcutaneous and intramuscular without affecting normal body functions and achieving satisfactory pain relief but with short duration of action, limiting their analgesic effectiveness [9]. The Cassic local anesthetics similar to NeoSTX, blocks axonal conduction by binding to the voltage-gated sodium channel but, instead of a highly selective pattern of classical local anesthestics, all of them block other sodium and non-sodium channels in a non-selective manner. On the other hand NeoSTX has a selective blocking effect over NA_V_ 1.2 [10,11]. This pharmacodynamical difference can explain the long lasting effect of NeoSTX that provides analgesia for over 24 h in animals and human volunteers [12,13].

In the last decade the interest in sodium blockers derived from marine toxins as a pharmacological alternative to traditional local anesthetics has increased due to their greater potency and longer lasting effect. Different clinical trials have used marine toxins in order to treat postoperative, cancer pain and neuromuscular disorders such as achalasia [14,15,16]. In addition to the administration of NeoSTX, bupivacaine or epinephrine potentiate the effect of subcutaneous NeoSTX, supporting the idea that this toxin is a new long acting local pain blocker with highly potential clinical use [13]. Nevertheless, there is not enough information about long term side effects and the effect of the route of administration in the acute toxicity of NeoSTX.

The present study using NeoSTX in an animal model is the first research about the chronic and acute toxicity by different routes of administration and shows direct evidence of the pharmacological profile of NeoSTX *in vivo* that allows to presume that its administration is safe. These findings also reveal possible future problems in error of administration and market presentation.

## 2. Results and Discussion

Shown below are bthe results of the determination of Lethal Dose (LD_50_), plasma concentration-time profile, as well as the effects of the administration of NeoSTX for 12 weeks (treatment period) and the next five weeks (recovery period) on body weight, food and water intake, weight of vital organs, hematological and biochemical parameters and histopathological assessment.

### 2.1. Acute Toxicity, Physical and Clinical Observations

In all cases, the majority of deaths were observed within the first 30 min. Few deaths occurred between 30 min and 1 h. No deaths occurred after 1 h. The highest dosages of the drug caused irregular respiration, cyanosis and death in the first 5 min by all routes of administration. The most common physical effect was decreased locomotor activity which disappeared after 2 h. Any clinical and physical signs appeared within seven days. Any area of necrosis or inflammation was noted at the injection site of those animals given parenteral doses and any evident injury to internal organs was remarked in survivor and dead animals.

**Figure 1:**
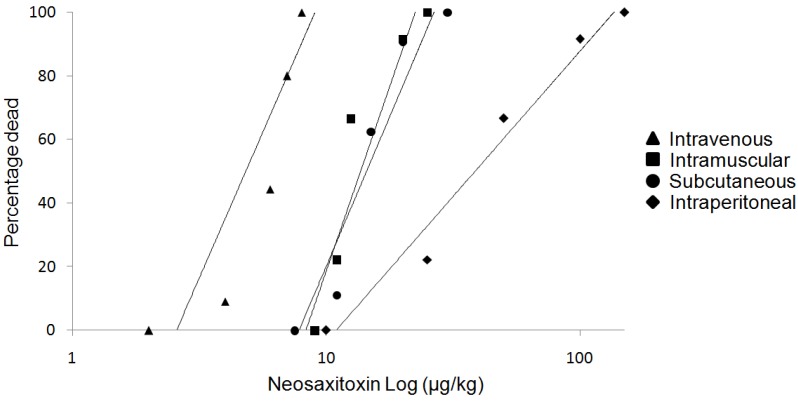
Dose-effect curves for neosaxitoxin given intraperitoneally, subcutaneously, intramuscularly and intravenously. Drug doses are shown on the logarithmically scaled horizontal axis, percentage deaths on the probit-scaled vertical axis. Note that the intramuscular and subcutaneous curves are close together.

Intramuscular (im) and subcutaneous (sc) LD_50_ doses for NeoSTX were not statistically different from each other (11.4 and 12.41 μg/kg, respectively). The intraperitoneal (ip) route presented the highest lethal dose reaching 30.35 μg/kg (*p* < 0.05). The intravenous (iv) route shown a LD_50_ of 6.06 μg/kg significantly lower than other routes (*p* < 0.05). The potencies in terms of LD_50_ were in the order iv < sc = im < ip (Figure 1, Table 1).

**Table 1:** Lethal (LD50) doses of neosaxitoxin by different routes (*n* = 15 per route).

	Intravenous	Intramuscular	Subcutaneous	Intraperitoneal
**LD_50_ (μg/kg)**	6.06	11.4	12.41	30.35
**Upper 95% CL**	8.14	13.69	16.15	46.51
**Lower 95% CL**	4.51	9.49	9.55	19.81

### 2.2. Plasmatic Concentration of NeoSTX

**Figure 2:**
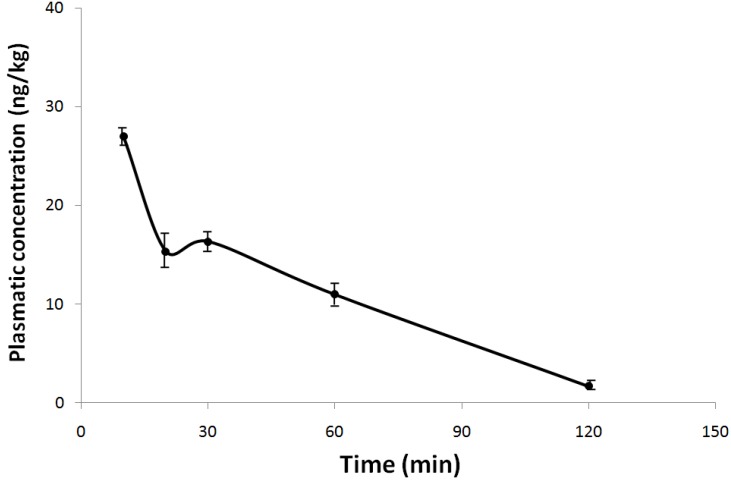
Mean plasma concentration-time curve of NeoSTX in rats after a single subcutaneous dose of 6 μg/kg. Each point represents the mean ± standard deviation (*n* = 15).

The mean plasma concentration-time profile of 6 μg/kg NeoSTX was evaluated when administered by the subcutaneous route (Figure 2). In order to study the elimination kinetics, blood samples were obtained at 10, 20, 30, 60 and 120 min, post administration of NeoSTX. After subcutaneous administration of NeoSTX in rats; plasma level of NeoSTX peaked at about 10 min with a Cmax value of 28 ng/kg. The Vd is 21.4 L, the elimination half life is approximately 46.4 min, elimination rate constant 0.896/h and a AUC 31.25 ng/L h.

Additionally, the plasmatic level of NeoSTX at the end of treatment period and recovery period was measured. At these times it was not possible to detect NeoSTX in plasma after 24 h administration.

### 2.3. Effect of NeoSTX on Body Weight, Food and Water Intake

Animals were randomized in five groups and daily treated by the subcutaneous route with saline solution (control), acetic acid 0.1% (solvent) and NeoSTX at doses of 1, 3 and 6 μg/kg (representing the pharmacologically active dose, three times the pharmacologically active dose and LD_50_ for the intravenous route). Rats were treated for 12 weeks (treatment period) and over the next five weeks did not receive treatment (recovery period).

**Figure 3:**
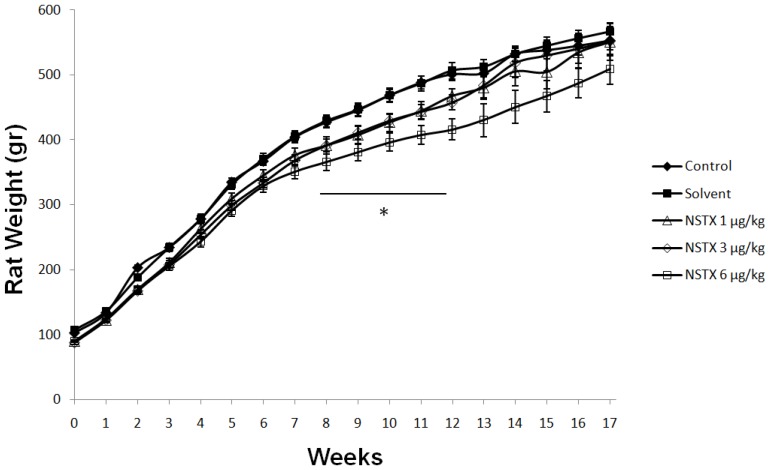
Effect of NeoSTX on body weight. Values are presented as mean ± SEM (*n* = 10). * *p* < 0.05 *vs.* control (one-way ANOVA followed by Bonferroni post test).

Compared to control and solvent groups, only the NeoSTX 6 μg/kg sc group showed significant reduction of body weight from week 8 to 12 (*p* < 0.05). Nevertheless, when the drug was suspended this group recovered the weight and did not present any differences with the control groups. On the other hand, significant reduction in weight was not seen with NeoSTX at doses of 1 μg/kg and 3 μg/kg (Figure 3).

**Figure 4:**
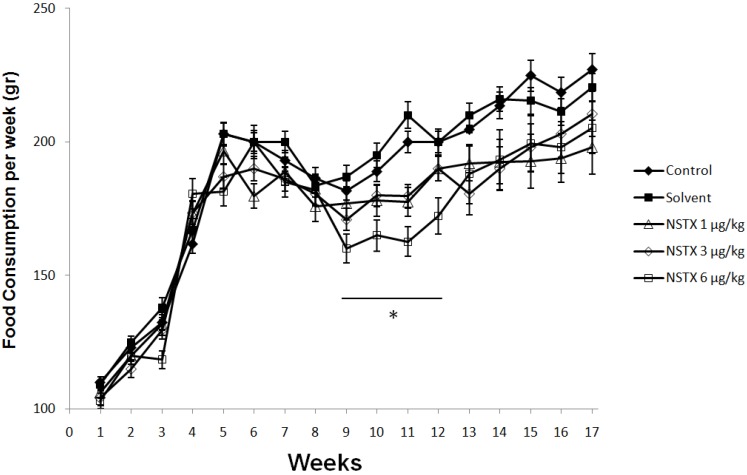
Effect of NeoSTX on daily food intake. Values are presented as mean ± SEM (*n* = 10 per group). * *p* < 0.05 *vs.* control (one-way ANOVA followed by Bonferroni post tests).

**Figure 5:**
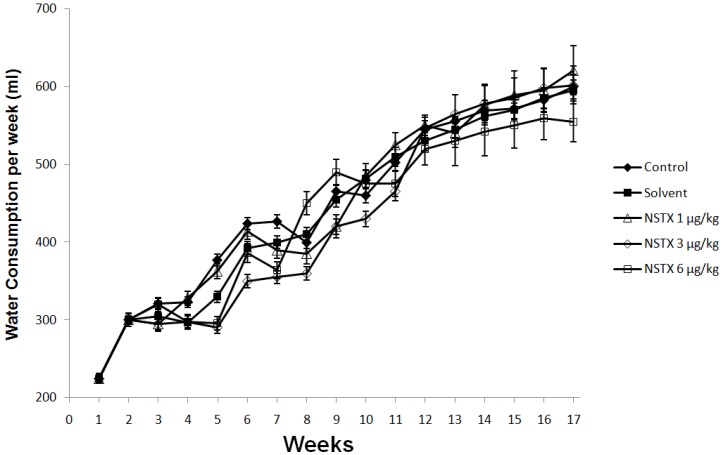
Effect of NeoSTX on weekly water intake. Values are presented as mean ± SEM (*n* = 7). * *p* < 0.01 *vs.* control (one-way ANOVA followed by Bonferroni post tests).

In respect to food intake, NeoSTX 6 μg/kg sc produced a significant effect (*p* < 0.05) on the average weekly food intake. At this dose, there were reductions in average weekly food intake in week 9–12 (Figure 4), however this difference was promptly reversed after 12 weeks on recovery period. On the other hand doses of NeoSTX, 1 μg/kg sc and 3 μg/kg sc, did not produce significant decrease in average weekly food intake compared to control and solvent groups in either treatment period or the recovery period (Figure 4). NeoSTX did not produce a change on average weekly water intake at any dose both in treatment and in recovery periods (Figure 5).

### 2.4. Effect on Weight of Vital Organs

NeoSTX did not produce any significant effect on the weight of vital organs at 12 weeks and at the end of the recovery study (Table 2 and Table 3).

**Table 2:** Effect of NeoSTX on weight of vital organs (per 100 g body weight) at 12 weeks (*n* = 5 per group).

	Heart	Spleen	Stomach	Liver	Lung	Kidney
**Control**	0.28 ± 0.02	0.26 ± 0.07	0.44 ± 0.04	3.04 ± 0.08	0.47 ± 0.08	0.69 ± 0.05
**Solvent**	0.30 ± 0.02	0.21 ± 0.01	0.42 ± 0.05	3.27 ± 0.44	0.42 ± 0.05	0.70 ± 0.03
**NeoSTX 1 μg/kg**	0.27 ± 0.01	0.21 ± 0.01	0.37 ± 0.02	2.97 ± 0.10	0.46 ± 0.02	0.64 ± 0.03
**NeoSTX 3 μg/kg**	0.30 ± 0.02	0.22 ± 0.02	0.47 ± 0.03	2.84 ± 0.19	0.46 ± 0.01	0.65 ± 0.07
**NeoSTX 6 μg/kg**	0.36 ± 0.04	0.24 ± 0.02	2.06 ± 0.11	3.47 ± 0.62	0.54 ± 0.07	0.75 ± 0.05

**Table 3:** Effect of NeoSTX on weight of vital organs (per 100 g body weight) at 17 weeks (*n* = 5 per group).

	Heart	Spleen	Stomach	Liver	Lung	Kidney
**Control**	0.27 ± 0.02	0.23 ± 0.06	0.42 ± 0.06	2.89 ± 0.16	0.44 ± 0.07	0.65 ± 0.03
**Solvent**	0.29 ± 0.04	0.20 ± 0.01	0.40 ± 0.07	3.13 ± 0.27	0.39 ± 0.05	0.66 ± 0.05
**NeoSTX 1 μg/kg**	0.25 ± 0.04	0.19 ± 0.03	0.33 ± 0.05	2.68 ± 0.49	0.41 ± 0.06	0.58 ± 0.12
**NeoSTX 3 μg/kg**	0.25 ± 0.03	0.18 ± 0.03	0.40 ± 0.08	2.40 ± 0.42	0.39 ± 0.05	0.57 ± 0.06
**NeoSTX 6 μg/kg**	0.36 ± 0.07	0.23 ± 0.03	0.45 ± 0.14	3.48 ± 0.86	0.44 ± 0.07	0.64 ± 0.11

### 2.5. Effect of NeoSTX on Hematological Parameters

NeoSTX did not produce any significant effect on hematological parameters at 12 weeks of NeoSTX administration (Table 4) and at 17 weeks after the recovery study (Table 5).

**Table 4:** Effect of NeoSTX on hematological parameters in rats at 12 weeks (*n* = 5 per groups).

	Control	Solvent	NSTX 1 μg/kg	NSTX 3 μg/kg	NSTX 6 μg/kg
**Hemoglobin (%)**	15.0 ± 2.3	15.4 ± 1.7	15.8 ± 3.4	16.4 ± 2.6	15.5 ± 1.9
**RBC Count (10^6^/μL)**	9.36 ± 0.30	9.40 ± 0.84	9.05 ± 0.22	9.23 ± 0.13	8.42 ± 1.30
**Hemotocrite (%)**	52.0 ± 1.4	52.9 ± 1.8	52.2 ± 2.2	52.3 ± 0.6	53.1 ± 2.1
**MCV (fL)**	55.5 ± 0.5	56.0 ± 4.3	57.6 ± 1.6	56.7 ± 0.6	55.1 ±2.3
**MCH (pg)**	16.9 ± 0.2	17.8 ± 0.3	17.1 ± 0.2	17.5 ± 0.4	17.2 ± 0.5
**MCHC (g/dL)**	30.4 ± 0.4	32.0 ± 2.6	31.4 ± 0.8	31.7 ± 0.2	32.4 ± 1.2
**Platelet count (10^3^/μL)**	1238 ± 360	1145 ± 407	995 ± 330	1050 ± 200	1090 ± 180
**WBC (10^3^/μL)**	3.9 ± 0.9	4.5 ± 2.3	5.3 ± 2.3	4.8 ± 0.7	4.0 ± 0.8
**Reticulocyte (%)**	0	0	0	0	0
**Neutrophils (%)**	12 ± 6	5 ± 2	7 ± 3	4 ± 2	7 ± 2
**Lynphocytes (%)**	88 ± 6	92 ± 2	92 ± 3	93 ± 3	91 ± 4
**Eosinophils (%)**	0	1 ± 1	1 ± 1	1 ± 1	2 ± 2
**Monocytes (%)**	0	1 ± 1	0	2 ± 2	0
**Basophils (%)**	0	0	0	0	0

**Table 5:** Effect of NeoSTX on hematological parameters in rats at 17 weeks (*n* = 5 per group).

	Control	Solvent	NeoSTX 1 μg/kg	NeoSTX 3 μg/kg	NeoSTX 6 μg/kg
**Hemoglobin (%)**	15.4 ± 2.6	14.7 ± 1.5	15.2 ± 2.4	15.7 ± 2.4	15.9 ± 2.3
**RBC Count (10^6^/μL)**	8.78 ± 0.50	9.32 ± 0.94	9.35 ± 0.78	9.32 ± 1.03	8.82 ± 1.55
**Hematocrit(%)**	51.9 ± 2.1	52.5 ± 1.2	52.7 ± 2.3	52.6 ± 1.1	53.2 ± 2.4
**MCV (fL)**	56.5 ± 1.5	56.5 ± 2.3	56.4 ± 1.8	56.3 ± 0.9	55.7 ± 1.8
**MCH (pg)**	17.5 ± 0.4	17.7 ± 0.6	17.0 ± 0.4	17.8 ± 0.4	17.1 ± 0.5
**MCHC (g/dL)**	31.4 ± 0.6	32.3 ± 1.7	31.7 ± 0.8	32.4 ± 1.1	32.2 ± 1.4
**Platelet count (10^3^/μL)**	1145 ± 242	1275 ± 322	1035 ± 240	1100 ± 220	1140 ± 245
**WBC (10^3^/μL)**	4.9 ± 1.5	4.4 ± 1.3	4.8 ± 1.9	5.4 ± 1.2	4.3 ± 1.2
**Reticulocyte (%)**	0	0	0	0	0
**Neutrophils (%)**	8 ± 4	6 ± 3	6 ± 2	5 ± 3	6 ± 3
**Lymphocytes (%)**	91 ± 7	92 ± 4	92 ± 3	92 ± 4	90 ± 3
**Eosinophils (%)**	0	1 ± 1	1 ± 1	1 ± 1	1 ± 1
**Monocytes (%)**	0	1 ± 1	1 ± 1	1 ± 1	1 ± 1
**Basophils (%)**	0	0	0	0	0

### 2.6. Effect of NeoSTX on Serum Biochemical Parameters

In the treatment period, there were significant increases (*p* < 0.05) in the concentration of total bilirubin in the group treated with NeoSTX at the dose of 6 μg/kg subcutaneous (sc) (0.38 ± 0.03 mg/dL) when compared with the control group (0.11 ± 0.02 mg/dL) (Table 6). Also, the direct bilirubin was augmented in the NeoSTX 6 μg/kg group sc (0.30 ± 0.07 mg/dL) in comparison to the control group (0.01 ± 0.02 mg/dL) at week 12 (Table 6). These changes on total bilirubin and direct bilirubin were reversed after five weeks of cessation of administration of NeoSTX (Table 7).

On the other hand, serum levels of GGT and SGOT were found to be slightly increased at 12 weeks against control group, however they normalized after drug suspension (Table 6 and Table 7).

**Table 6:** Effect of NeoSTX on serum biochemical parameters at 12 weeks (*n* = 5 per group). * *p* < 0.05 *vs.* control.

	Control	Solvent	NeoSTX 1 μg/kg	NeoSTX 3 μg/kg	NeoSTX 6 μg/kg
**Glucose (mg/dL)**	326.2 ± 82.4	367.8 ± 59.95	289.6 ± 45.23	303.8 ± 47.21	307.8 ± 117.78
**Ureic Nitrogen (mg/dL)**	15.5 ± 2.1	18.8 ± 1.17	17.4 ± 1.85	15.6 ± 1.6	18.4 ± 1.4
**Ureic acid (mg/dL)**	1.02 ± 0.25	1.12 ± 0.44	0.88 ± 0.44	1.04 ± 0.32	4.5 ± 4.00
**Total Protein (g/dL)**	6.42 ± 0.12	6.26 ± 0.36	6.46 ± 0.28	6.44 ± 0.36	6.65 ± 0.37
**Albumin (g/dL)**	3.28 ± 0.21	3.2 ± 0.16	3.28 ± 0.22	3.38 ± 0.15	3.59 ± 0.24
**Total Cholesterol (mg/dL)**	58.2 ± 6.58	61.2 ± 5.71	72.4 ± 13.66	64.8 ± 7.76	68.6 ± 5.38
**HDL (mg/dL)**	35.4 ± 5.78	39.2 ± 3.41	46.8 ± 6.23	41.2 ± 5.27	46.6 ± 8.69
**LDL (mg/dL)**	7.72 ± 5.06	14.28 ± 16.98	9.42 ± 5.63	12.76 ± 4.59	10.08 ± 5.79
**Triglycerides (mg/dL)**	75.8 ± 28.13	132.5 ± 107.88	107.4 ± 70.43	54.2 ± 20.88	91.2 ± 40.36
**LDH (U/L)**	2383.8 ± 760.51	2356.6 ± 1024.64	2475.2 ± 528.45	2302.6 ± 727.33	4015.5 ± 2497.87
**Creatinine (mg/dL)**	0.52 ± 0.04	0.54 ± 0.05	0.54 ± 0.05	0.46 ± 0.05	0.54 ± 0.08
**Total Bilirubin (mg/dL)**	0.11 ± 0.02	0.13 ± 0.02	0.14 ± 0.02	0.13 ± 0.02	0.38 ± 0.03 *
**Direct Bilirubin (mg/dL)**	0.01 ± 0.02	0.02 ± 0.04	0.03 ± 0.04	0.02 ± 0.02	0.30 ± 0.07 *
**Alkaline Phosphatase (U/L)**	206.4 ± 56.57	238.2 ± 62.31	293.4 ± 30.72	292.2 ± 68.38	237.4 ± 67.93
**GGT (U/L)**	7.2 ± 3.53	7.4 ± 0.84	7.5 ± 0.87	8.2 ± 4.17	12.5 ± 2.75 *
**SGOT (UI/dL)**	91.2 ± 8.45	88.6 ± 12.12	95.7 ± 10.92	81.2 ± 11.07	142.8 ± 15.42 *
**SGPT (UI/dL)**	67.8 ± 6.96	60.9 ± 6.93	61.0 ± 4	75.4 ± 12.81	80.2 ± 15.32

**Table 7:** Effect of NeoSTX on serum biochemical parameters at 17 weeks (*n* = 5 per group).

	Control	Solvent	NeoSTX 1 μg/kg	NeoSTX 3 μg/kg	NeoSTX 6 μg/kg
**Glucose (mg/dL)**	334.7 ± 61.3	352.5 ± 45.67	297.5 ± 53.2323	335.8 ± 52.32	325.8 ± 52.46
**Ureic Nitrogen (mg/dL)**	16.3 ± 1.73	17.6 ± 1.49	15.9 ± 1.57	18.4 ± 2.1	18.1 ± 1.6
**Ureic acid (mg/dL)**	0.92 ± 0.42	1.22 ± 0.54	0.96 ± 0.51	1.10 ± 0.3	1.5 ± 0.57
**Total Protein (g/dL)**	6.32 ± 0.24	6.36 ± 0.26	6.42 ± 0.26	6.46 ± 0.5	6.35 ± 0.47
**Albumin (g/dL)**	3.30 ± 0.19	3.24 ± 0.17	3.28 ± 0.19	3.32 ± 0.22	3.29 ± 0.20
**Total Cholesterol (mg/dL)**	59.3 ± 7.38	62.6 ± 6.52	64.4 ± 6.35	63.8 ± 5.8	62.8 ± 6.18
**HDL (mg/dL)**	38.6 ± 4.52	43.1 ± 4.43	42.9 ± 5.36	43.5 ± 6.10	41.8 ± 6.12
**LDL (mg/dL)**	10.53 ± 4.34	12.58 ± 6.33	10.5 ± 5.42	11.34 ± 4.87	10.54 ± 4.90
**Triglycerides (mg/dL)**	93.5 ± 23.4	102.3 ± 36.4	90.6 ± 22.4	76.8 ± 25.66	105.2 ± 32.10
**LDH (U/L)**	2525.4 ± 665.50	2248.6 ± 735.42	2160.8 ± 460.76	2650.2 ± 643.55	2540.9 ± 580.32
**Creatinine (mg/dL)**	0.53 ± 0.05	0.54 ± 0.04	0.52 ± 0.04	0.51 ± 0.05	0.53 ± 0.05
**Total Bilirubin (mg/dL)**	0.11 ± 0.02	0.12 ± 0.01	0.11 ± 0.01	0.10 ± 0.03	0.12 ± 0.03
**Direct Bilirubin (mg/dL)**	0.02 ± 0.02	0.03 ± 0.02	0.02 ± 0.03	0.01 ± 0.02	0.03 ± 0.02
**Alkaline Phosphatase (U/L)**	226.8 ± 48.37	242.9 ± 54.35	248.9 ± 42.52	225.2 ± 45.55	235.6 ± 49.76
**GGT (U/L)**	7.2 ± 1.51	7.3 ± 1.22	7.4 ± 0.91	7.8 ± 2.45	7.4 ± 1.73
**SGOT (UI/dL)**	89.5 ± 11.55	91.6 ± 11.83	96.4 ± 10.65	88.9 ± 10.88	95.8 ± 12.45
**SGPT (UI/dL)**	65.4 ± 5.87	65.2 ± 4.22	62.3 ± 4.55	68.2 ± 5.28	70.1 ± 8.77

### 2.7. Effect of NeoSTX on Serum Electrolyte

In this study, serum concentrations of electrolytes were measured at treatment and recovery periods, but there were no changes over these parameters (Table 8 and Table 9).

**Table 8:** Effect of NeoSTX on serum electrolytes at 12 weeks (*n* = 5 per group).

	Control	Solvent	NeoSTX 1 μg/kg	NeoSTX 3 μg/kg	NeoSTX 6 μg/kg
**Calcium (mg/dL)**	9.90 ± 0.41	9.88 ± 0.28	9.92 ± 0.14	9.84 ± 0.17	10.26 ± 0.81
**Phosphorus (mg/dL)**	7.33 ± 0.94	7.66 ± 0.69	7.59 ± 0.59	7.45 ± 0.72	9.71 ± 3.72
**Sodium (mEq/L)**	142.1 ± 1.41	140.8 ± 2.56	141.2 ± 2.13	142.4 ± 0.84	143.4 ± 4.84
**Potassium (mEq/L)**	5.32 ± 1.11	5.32 ± 0.25	5.35 ± 0.36	4.96 ± 0.57	7.66 ± 2.81
**Chloride (mEq/L)**	108.6 ± 1.28	106.2 ± 2.99	106.4 ± 1.59	107.8 ± 0.98	87.6 ± 37.90

**Table 9:** Effect of NeoSTX on serum electrolytes at 17 weeks (*n* = 5 per group).

	Control	Solvent	NeoSTX 1 μg/kg	NeoSTX 3 μg/kg	NeoSTX 6 μg/kg
**Calcium (mg/dL)**	9.85 ± 0.43	9.78 ± 0.38	9.9 ± 0.24	9.82 ± 0.17	9.82 ± 0.31
**Phosphorus (mg/dL)**	7.44 ± 0.62	7.45 ± 0.59	7.60 ± 0.67	7.52 ± 0.62	7.7 ± 0.72
**Sodium (mEq/L)**	142.3 ± 1.75	141.5 ± 1.86	142.1 ± 2.05	141.8 ± 1.71	141.4 ± 2.10
**Potassium (mEq/L)**	5.29 ± 0.65	5.31 ± 0.42	5.32 ± 0.47	5.33 ± 0.66	5.35 ± 0.93
**Chloride (mEq/L)**	107.5 ± 1.45	106.8 ± 1.95	107.1 ± 1.7	106.2± 1.11	106.6 ± 1.90

### 2.8. Effect of NeoSTX on Histopathological Presentations and Macro Anatomical Features

**Figure 6:**
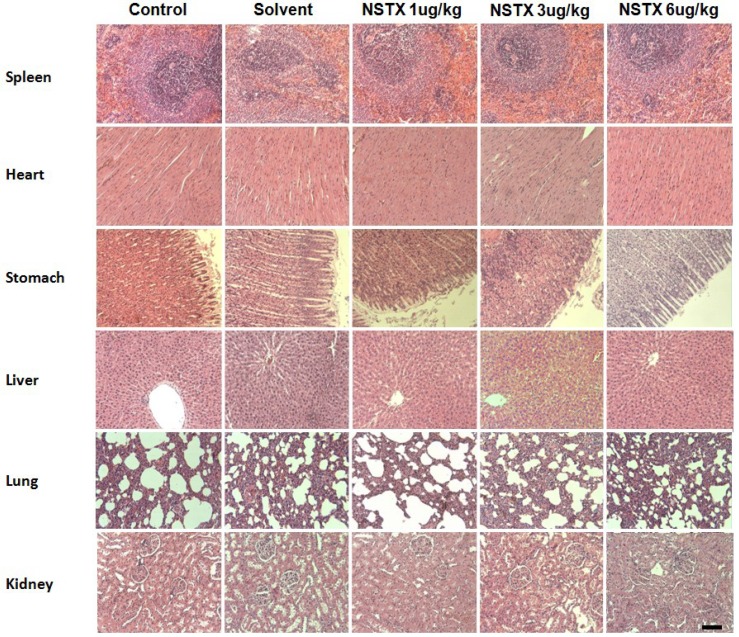
Effect of NeoSTX on histologic structure at 12 weeks. Selected microphotographs 10×, HE (scale bar = 200 µm).

At the end of treatment period and recovery periods, no significant histopathological changes were observed during autopsy in all treatment groups. Figure 6 shows histological results at 12 weeks. The spleen was not congested in all treatment groups. The heart was normal in all the treatment groups. The cardiac myocytes were arranged in interlacing and parallel array. Their nuclei were spindle shaped and elongated. In the liver there were no adverse histopathological effects observed in the control and NeoSTX treatment groups. The liver appeared normal with preserved hepatic architecture, hepatocytes arranged as radial plates, and having eosinophilic cytoplasm and central nuclei. No cytoplasmic inclusions were seen and no portal inflammation. The lungs were normal in all the treatment groups. The alveolar air spaces were surrounded by interstitium containing few blood vessels and inflammatory cells. In the kidney there were no adverse histopathological presentations observed in all the treatment groups. Normocellular glomerular tufts were displayed on a background containing tubules. No necrosis was observed Histological study at 17 weeks did not show any difference between control and treatment groups.

### 2.9. Mortality

In the chronic toxicity study, there were no deaths in the control group, the solvent group and the NeoSTX (1, 3, 6 μg/kg subcutaneous) treatment groups within treatment or recovery periods.

## 3. Discussion

The scientific interest in the search for new drugs for pain management has generated in the last decade increased studies with drugs derived from marine toxins such as NeoSTX [15]. Currently there are ongoing Phase III clinical studies of NeoSTX, therefore the study of the effects of acute and chronic administration of NeoSTX as well as the study of its toxicity have importance not only from an environmental point of view, but also it is of vital importance in the monitoring of possible adverse reactions in humans in future preparations that will be sold on the market.

Our acute toxicity study has shown that NeoSTX produces a rapid respiratory failure in the first hour, but delayed deaths (>1 h after the dose) were not observed after parenteral doses and after subcutaneous administration. These findings with regard to the manifestations of toxicity and the cause of death were consistent with those reported by Kohane [17] on NeoSTX, and were similar to the acute effects reported for chemically related drugs such as tetrodotoxin and saxitoxin [17,18,19,20]. The clinical manifestation of acute toxicity and time of death are consistent with plasma levels of NeoSTX. The most likely mechanism to explain the respiratory failure is the affinity of NeoSTX for the adult rat skeletal muscle Na^+^ channel (NA_V_ 1.2) that induces muscle weakness and diaphragmatic paralysis [20]. We also found differences between our study and previous studies. Munday [21], found a LD_50_ of 8.9 nmol/kg (2.8 μg/kg) intraperitoneally which is divergent to our finding of 30.35 μg/kg intraperitoneally. An explanation for the divergence in these results is the use of mouse and rats respectively. In rats, Kohane [17] determined the therapeutic index and lethality of NeoSTX. He reported the LD_50_ of 14 nmol/kg (4.1 μg/kg) intramuscularly for NNeoSTX which is a lower dose than our result of 11.4 μg/kg by the same route of administration. We assume that the differences in these studies were, in the study mentioned, due to injecting the same volume (100 μL) at higher concentration regardless of the animal’s weight. On the other hand the administration site was different; Kohane [17] injected the drug posteromedial to the greater trochanter very close to the bone. In this study it was injected in the quadriceps femoris muscles with a lesser concentration of drug. These findings suggest that more diluted administration of NeoSTX increases the LD_50_ and therefore the safety.

A small number of clinical trials of NeoSTX have been completed and all of them by subcutaneous routes [12,15], and NeoSTX should have a promising utility in postoperative analgesia. Nevertheless the characterization of the LD_50_ at different routes has not been completed yet, and this would be useful in the safety description of the drug. In this study, our findings have shown no statistical differences between intramuscular LD_50_ and subcutaneous LD_50_, on the other hand the intraperitoneal LD_50_ was approximately three times higher than the intramuscular and subcutaneous LD_50_. This pharmacological profile of the NeoSTX allows us to presume that its administration is safe, if avoiding adverse drug reactions due to errors in subcutaneous administration and incidental spill into the peritoneal cavity.

In the chronic toxicity study, there were no death events in the control groups, solvent group and NeoSTX (1, 3, 6 μg/kg) by the subcutaneous route within the treatment period and recovery period. These doses represent the pharmacologically active dose used in the clinical trial [15], three times the pharmacologically active dose and LD_50_ for the intravenous route, respectively.

Solely the daily administration of 6 μg/kg NeoSTX showed a significant reduction in body weight from week 8 to 12 and average weekly food intake from week 9 to 12. Similarly we observed a significant increase in total and direct bilirubin during chronic treatment with NeoSTX as well as increased GGT and SGOT, all of which normalized after drug suspension. Other changes were not shown in the studied biochemical and histological parameters. These changes are compatible to cholestasis pattern; cholestasis may either result from a functional defect in bile formation at the level of the hepatocyte or from impairment in bile secretion and flow at the bile level [22]. The bile secretion plays a pivotal role in the liver physiology which is metabolized by the uridine diphosphate (UDP)-glucuronosyltransferases (UGTs), which is a key enzyme in human detoxication of endobiotics and xenobiotics [23,24,25]. Garcia [26,27] has recently demonstrated that oxidation and glucuronidation are initial detoxification reactions for excretion of NeoSTX and possibly such as morphine, acetaminophen and estradiol-17β which are glucuronized in liver and are well known for their potential hepatotoxicity; the chronic exposure of NeoSTX may induce changes in expression and function of hepatobiliary proteins [28]. On the other hand, the glucuronidization of NeoSTX is an important step in renal clearance; a specific defect in this enzymatic pathway may induce lower toxicity thresholds. Moreover, another explanation of the cholestasis pattern may be the reduction of food consumption, which induces a condition of fasting and it is well known that this factors in the genesis of cholestasis pattern in humans [29], as well as in animals like monkeys, horses, and rats [30,31,32]. Nevertheless, the changes seen in the treatment period were reversed after drug suspension.

## 4. Experimental Section

### 4.1. Animals

Male Sprague Dowley rats were used in the acute and chronic study, on average weighing 100 g. Experiments were performed according to the principles of the OECD Guideline 425 [33] and current guidelines for the care of laboratory animals and ethical guidelines approved by the Animal Care and Use Committee of the Faculty of Medicine, University of Chile (CBA# 0632 FMUCH). Animals were obtained from animal facilities of the Faculty of Medicine. They were housed on a 12 h light-dark cycle at 22 ± 2 °C and with access to food and water *ad libitum*.

### 4.2. Acute Toxicity Study

Acute toxicity study rats were acclimatized to the laboratory for at least 2 h before testing, used only once during the protocol and sacrificed 7 days after the administration of the drug. For the Lethal Dose curves, the minimal number of animals was used in each of the four dosage groups to establish the Lethal Dose 50 (LD_50_) in all routes of studied administration (no more than 15 animals). Linear regression analysis of the log dose-response curves allowed the calculation of the LD_50_. NeoSTX was provided by Proteus S.A. (Santiago, Chile). Each animal was used only once, and received only one dose of the drugs tested. All drugs were freshly prepared by dissolving them in normal saline, and administered intraperitoneally, intramuscular, subcutaneous and intravenous. The authors performed all observations in a randomized and blind manner. After dosing, rats were housed in groups of two. NeoSTX was administered at constant volume of 10 mL/kg in intraperitoneal, intramuscular and subcutaneous routes; however intravenous was administrated at a constant volume of 5 μL. LD_50_ was estimated by the log dose-probit analysis method [34] based on mortality recorded within 7 days.

### 4.3. Chronic Toxicity Study

A total of 50 rats were randomly allotted to five groups housed separately in polypropylene cages. The animals were daily treated by the subcutaneous route with saline solution (control), acetic acid 0.1% (solvent) and NeoSTX at doses of 1, 3 and 6 μg/kg (representing the pharmacologically active dose, three times the pharmacologically active dose and LD_50_ for intravenous route).

Rats were weighed weekly and observed for behavioral changes, feeding and drinking habits, and general morphological changes. At the end of the 12 weeks of treatment period, from each group five animals were selected randomly and anesthetized with 100 mg/kg pentobarbital ip. Blood samples were collected from rats by cardiac puncture into EDTA (ethylenediamine-tetra acetate) sample bottles for hematological analysis and into plain sample bottles for serum generation for biochemical analysis. Serum was obtained after allowing blood to coagulate for 30 min. and centrifugation. After euthanizing the experimental animals, vital organs including the heart, lungs, spleen, kidneys, testicles and stomach were harvested, carefully examined for gross lesions, and weighed. The weight of each organ was standardized to 100 g body weight of each animal. The organs were preserved in 10% formol-saline for histopathological assessment. Mortality in each treatment group was recorded during the course of the 12 week treatment period of NeoSTX and recovery period of 5 weeks in which animals were not treated with the drug. At the end of the recovery period, rats were sacrificed and assays conducted at the end of the treatment period.

### 4.4. Hematological Analysis

Blood samples were analyzed using established procedures and with an automated hematology analyzer. Parameters evaluated included hemoglobin (Hb), red blood cell count (RBC), packed cell volume (PCV), mean red cell volume (MCV), mean cell hemoglobin (MCH), mean cell hemoglobin concentration (MCHC), platelet count, white blood cell (WBC) count and differential white blood cell count.

### 4.5. Biochemical Analysis

Serum samples were analyzed for glucose, ureic nitrogen, uric acid, total protein, albumin, total cholesterol, high density lipoprotein (HDL), low density lipoprotein (LDL), triglycerides (TG), LDH, creatinine, gamma-glutamyltransferase (GGT), serum glutamic oxaloacetic transaminase (SGOT), serum glutamic-pyruvic transaminase (SGPT), total bilirubin, and direct bilirubin using Roche and Cobas commercial kits and a Roche/Hitachi 904 automated analyzer (Roche Diagnosis, Hitachi, Manheim, Germany). Serum electrolyte concentration was determined by established methods; sodium and potassium concentration by flame photometry, chloride and bicarbonate concentration by titrimetric methods, and calcium concentration by the cresol phthalein complexone method [33,34,35,36].

### 4.6. Histopathological Assessment

Animals were euthanized at 12 and 17 weeks and organs/tissues were carefully examined macroscopically and gross lesions were recorded. During necropsies, the following organs were weighed separately: heart, lungs, kidneys, liver, spleen and stomach. The tissues obtained from experimental animals fixed in 10% formol–saline were dehydrated in graded alcohol, embedded in paraffin, and cut into 4–5 μm thick sections. Hematoxylin-eosin was used to stain the sections for photomicroscopic assessment using a Model N-400ME photomicroscope (CEL-TECH Diagnostics, Hamburg, Germany). Slides were examined using the ×40, ×100, and ×400 objectives.

### 4.7. Pharmacokinetic Study

Fifteen rats received a single subcutaneous dose, 6 μg/kg of NeoSTX. After dosage, blood samples were collected into heparinized tubes at 10, 20, 30, 60, 120 min. Plasma was isolated from the blood samples by centrifugation. Paralytic shellfish toxins were determined under the previously described conditions, using ion pairing chromatography with post-column derivatization [37]. Briefly, 20 μL treated samples were injected (model 7725i with a 20-μL loop, Rheodyne, Rohnert Park, CA, USA) to a silica-base reversed phase column (Agilent Zorbax Bonus RP, 3.5 μm, 4.6 mm × 150 mm; Agilent Technologies, Santa Clara, CA, USA). A mobile phase (Solvent A) 11 mM heptane sulfonate, 5.5 mM H_3_PO_4_, pH 7.1; (Solvent B) 11 mM heptane sulfonate, 16.5 mM H_3_PO_4_, 11.5% MeCN, pH 7.1, at a flow rate of 0.8 mL/min was used for detection and quantitation of NeoSTX. Gradient conditions used were 100% A at 0–8 min, 100% B at 8.01–19 min and 100% A at 19.01–30 min. The elution from the column was continuously mixed 100 mM H_3_PO_4_, 5 mM H_5_IO_6_ pH 7.8 at 0.4 mL/min heated at 85 °C with 1 mL knitted coil (0.5 mm i.d., 10 m long) and then mixed with 0.75 M HNO_3_ at 0.4 mL/min before entering the fluorescent detector (Agilent Technologies, Kirkland, PQ, Canada). The fluorescent detector was set at an excitation wavelength of 330 nm and an emission wavelength of 390 nm; run time, 30 min. The LC system was equilibrated for 320 min at a column oven temperature of 30–40 °C with 100% solvent A flowing at 0.8 mL/min, and a flow of 0.4 mL/min for the oxidant and acid. An Agilent 1100 quaternary solvent delivery system liquid chromatograph apparatus with an Agilent 1200 spectrofluorometric detector was used (Agilent Technologies, Kirkland, PQ, Canada). Data acquisition and data processing were performed with Agilent ChemStation Chromatography Data System. Toxins were identified and quantified by comparing their retention times (*R*_t_) and fluorescent response with standard solutions. Peak to concentration linear correlation, with an *r*^2^ coefficient of 0.99, was found. The limit of detection was set to a signal to noise ratio of 3:1. Standards were certified reference materials obtained from the National Research Council Canada, Institute for Marine Biosciences. Toxin internal standards are normally run as routine control, starting in the morning before sample injections, at midday in the working day and after the last injected sample as a final control of the chromatographic run. PK Solver software was employed to analyze the plasma concentration-time data with non-compartimental analysis. The area under the concentration-time curve (AUC), elimination half-life time (*T*_1/2_), volume of distribution (Vd), plasma clearance (Cl), elimination rate constant (Ke) and peak concentration (*C_max_*) of the drug were all obtained.

### 4.8. Statistical Analysis

Results are expressed as mean ± SEM. Data analysis was carried out using one-way ANOVA followed by Bonferroni post tests using GraphPad Prism 5 (GraphPad Software Inc., La Jolla, CA, USA). Significance was considered at values of *p* < 0.05.

## 5. Conclusions

In conclusion, the acute and chronic toxicity of NeoSTX was carefully evaluated over a wide range of doses and administration routes. The acute toxicity of NeoSTX is influenced by the rapidity of absorption from parenteral administration of the drug and its rapid clearance. On the other hand in the chronic toxicity study, our results show that solely the highest studied dose induced a reduction of weight and food consumption in weeks 8–12 accompanied by a cholestasic pattern at the end of the treatment period. Changes described above were completely normalized with the drug suspension. This finding supports the safe pharmacological profile of NeoSTX and also allows important clinical considerations for future clinical trials and use in patients.
